# Three-Dimensional Anatomically Pre-Contoured Locking Plate for Isolated Weber B Type Fracture

**DOI:** 10.3390/jcm10132976

**Published:** 2021-07-02

**Authors:** Jahyung Kim, Dong-Il Chun, Sung-Hun Won, Tae-Hong Min, Young Yi, Suyeon Park, Min-Soo Cho, Jaeho Cho

**Affiliations:** 1 Department of Orthopaedic Surgery, Armed Force Gangneung Hospital, Gangneung 25422, Korea; hpsyndrome@naver.com; 2 Department of Orthopaedic Surgery, Soonchunhyang University Seoul Hospital, Seoul 04401, Korea; orthochun@gmail.com (D.-I.C.); orthowon@gmail.com (S.-H.W.); minth916@gmail.com (T.-H.M.); 3 Department of Orthopaedic Surgery, Inje University Seoul Paik Hospital, Seoul 04551, Korea; 20vvin@naver.com; 4 Department of Biostatistics, Soonchunhyang University Seoul Hospital, Seoul 04401, Korea; suyeon1002@schmc.ac.kr; 5 Department of Orthopaedic Surgery, Chuncheon Sacred Heart Hospital, Hallym University, Chuncheon 24253, Korea; minso1017@naver.com

**Keywords:** patient specific, orthopedic implants, ankle fracture, locking plate

## Abstract

We aimed to evaluate the functional and radiographic outcomes of a three-dimensionally (3D) pre-contoured lateral locking plate fixation for isolated Weber B type fractures and to evaluate the necessity of an interfragmentary lag screw in the use of the plate. Patients who underwent surgery for isolated Weber B type fracture were divided into two groups: 41 patients treated with the 3D plate and lag screw (Group A) and 31 patients treated with the 3D plate only (Group B). The included patients were evaluated regarding the functional and radiographic outcomes. According to the McLennan and Ungersma criteria, the majority of patients showed good or fair outcomes in both groups. Comparing the two groups, Group B showed better functional outcomes (*p* < 0.0046), while no difference between the two groups was found in terms of the radiographic outcomes (*p* = 0.143). The operation time was significantly shorter in Group B (*p* < 0.001) and the time to bony union was within 14 months in all patients with no significant difference between the two groups (*p* = 0.0821). No postoperative complication was observed in both groups. In conclusion, the use of a 3D pre-contoured lateral locking plate fixation for isolated Weber B type fractures demonstrated satisfactory functional and radiographic outcomes, regardless of lag screw insertion.

## 1. Introduction

Ankle fractures are one of the most frequently encountered injuries by orthopaedic surgeons. Among these fractures, isolated unimalleolar distal fibular fractures are known to account for more than 50% of all ankle fractures [1]. These fractures generally result from rotational injuries and form a spiral or oblique fracture line originating at the level of tibial syndesmosis. Based on radiographic findings, such fractures correspond to Weber B type fractures or Lauge–Hansen supination external rotation (SER) injuries [2,3].

The treatment options for an isolated Weber B type fracture vary, and surgical stabilization is usually recommended to prevent ankle joint subluxation and promote biomechanical recovery in an unstable fracture [4,5]. In terms of surgical fixation devices for an isolated Weber B type fracture, lag screws in combination with locking plates are preferred currently [6]. With the development of a pre-contoured locking plate, however, studies showed that plate itself can provide sufficient fixation power beyond its role of neutralizing the fixation accomplished by lag screws [7,8].

**Figure 1:**
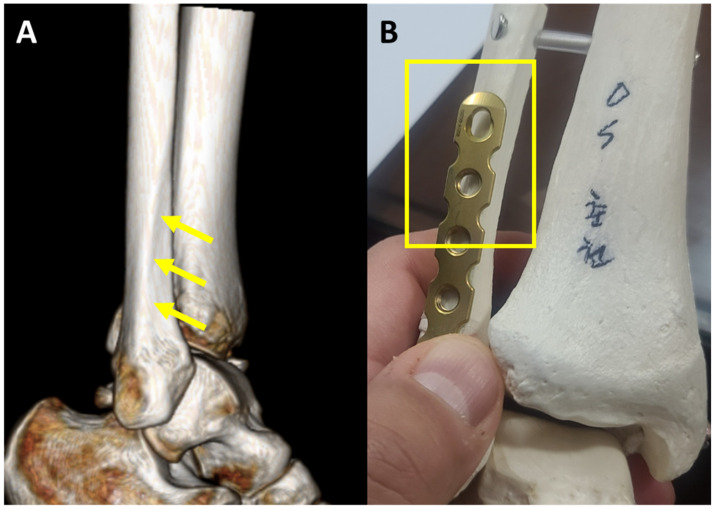
(**A**) Morphology of distal fibula presenting an interosseous crest (arrows), dividing anterior and lateral surface of the fibula. (**B**) Proximal portion of the two-dimensionally contoured plate is not fit on the bone surface (square box).

Most of the pre-contoured locking plates widely used are bent two-dimensionally. However, it is noteworthy that the three-dimensional morphology of the distal fibula does not appear to be reflected in these plates. In other words, the proximal portion of the two-dimensionally pre-contoured plates cannot be fit appropriately to the bone contour owing to the interosseous crest within the fibula (Figure 1). As a result, a lack of contact between the proximal plate and the fibular body may result in insufficient stability of the fracture fixation [9].

Based on these findings, we hypothesized that with the use of a three dimensional, anatomically pre-contoured lateral locking plate considering the morphology of distal fibula, the fracture site could be fixed properly, even in the absence of interfragmentary lag screws. As a result, we aimed to evaluate the postoperative functional and radiographic outcomes of a three-dimensionally pre-contoured lateral locking plate fixation for isolated Weber B type fractures. In addition, we attempted to evaluate the necessity of an interfragmentary lag screw with the use of the plate.

## 2. Materials and Methods

This retrospective multi-centre study was approved by the medical ethics committee at our institution (Institutional Review Board number: CHUNCHEON 2021-01-007-001), and written informed consent for publication of this report was obtained from all included patients. From November 2015 to January 2020, a total of 188 consecutive patients who underwent surgery for isolated Weber B type fractures were enrolled. 

An isolated Weber B type fracture was defined as a lateral malleolar fracture that originates at the level of the syndesmosis without concurrent unstable syndesmotic injury that requires surgical fixation [1]. Syndesmotic instability was evaluated intraoperatively through the fluoroscopic lateral stress test (i.e., the Cotton test) [10]. Two orthopaedic surgeons on our team with more than ten years of clinical experience in foot and ankle surgery performed the operations using the same operative technique. 

**Figure 2:**
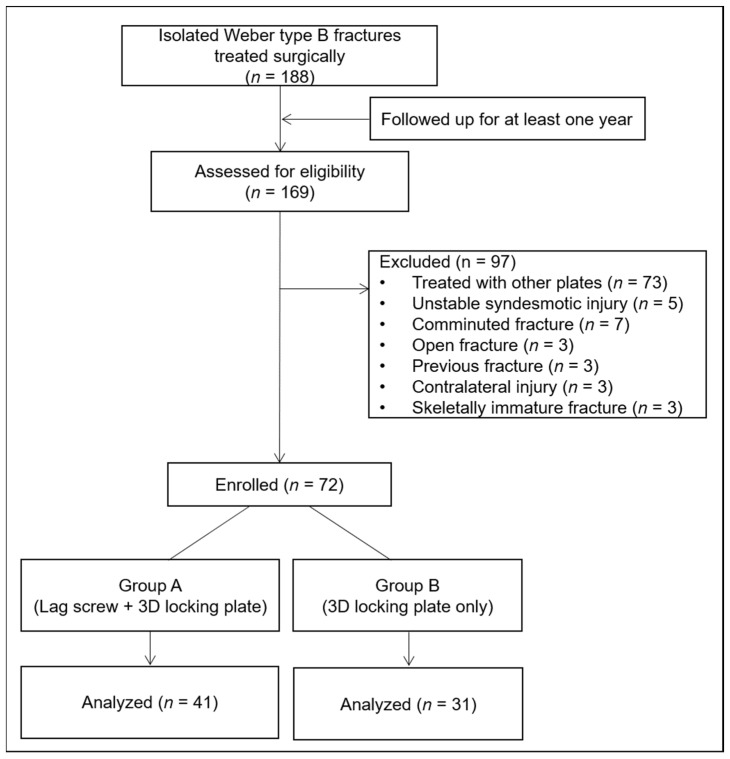
Flow chart of study group selection by means of the inclusion and exclusion criteria. 3D = three-dimensional.

Of the enrolled patients, those who were followed up for at least one year were included. Patients treated with other types of plate and those with concomitant unstable ligament injury, comminuted fracture, open fracture, previous fracture of the involved limb, injuries of the contralateral limb, and skeletally immature fracture were excluded. Eventually, 72 patients were included in this study (Figure 2). Patient demographic data, including age, sex, body mass index, operation time, and follow up period, were analysed through medical record reviews.

**Figure 3:**
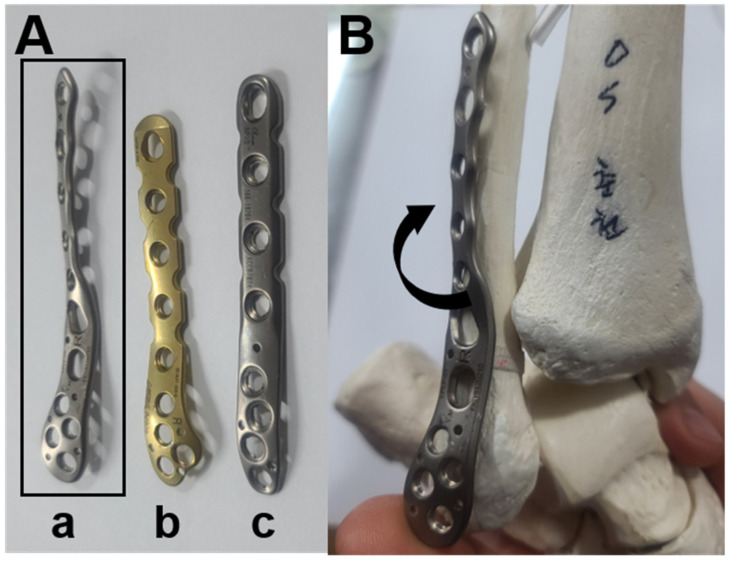
(**A**) Morphology of the three-dimensionally pre-contoured locking plate (**a**) compared to two-dimensionally pre-contoured locking plates (**b**,**c**). (**B**) Considering the interosseous crest of the fibula, the proximal portion of the plate is twisted posteriorly and can be fit anatomically on the bone contour.

Based on the different fixation device used, the patients were divided into two groups: Group A (*n* = 41): patients treated with the use of an interfragmentary lag screw and a pre-contoured locking plate (Taeyeon Med, Wonju, Korea), and Group B (*n* = 31): patients treated only with the use of a pre-contoured locking plate. The plate was three-dimensionally contoured based on the anatomy of the fibula. Considering the interosseous crest of the fibula, the proximal portion of the plate is twisted posteriorly, in order to be fit anatomically on the bone contour (Figure 3). 

### 2.1. Surgical Procedures

Following an open reduction of the fracture using a reduction clamp, one 3.5-mm cortical screw was inserted perpendicular to the fracture line anteriorly to posteriorly in Group A. After the lag-screw fixation, the fracture site was additionally fixed with a three-dimensionally pre-contoured locking plate applied on the external side, adjusting it to the morphology of the fibula. 

In Group B, the fracture site was temporarily maintained without withdrawing the reduction clamp or by percutaneously inserting two Kirschner wires posterodistally to anteroproximally, perpendicular to the fracture line [11]. In the wake of temporary fixation, the plate was applied to the fibula in the same manner. In terms of plate fixation, a distal locking screw was first fixed on the distal part of the fracture. Then, a cortical screw was inserted on the proximal part of the fracture. 

**Figure 4:**
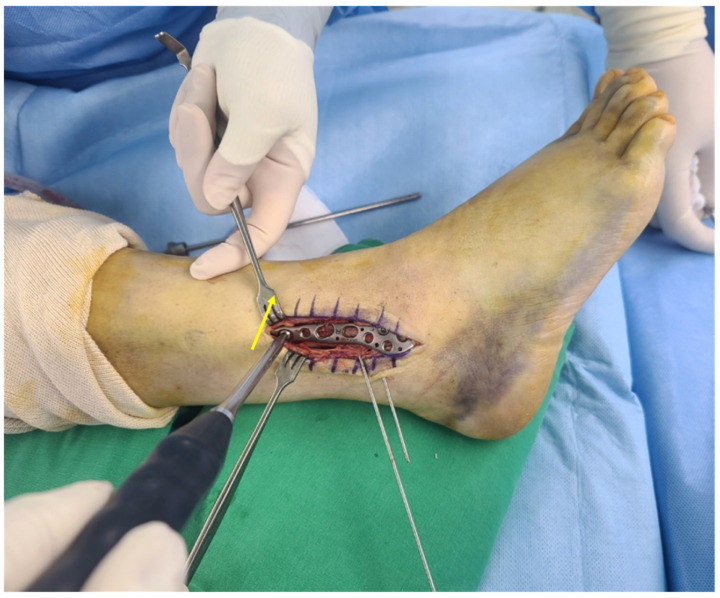
Intraoperative image showing the cortical screw insertion on the proximal part of the fracture. As the proximal portion of the plate is bent posteriorly, posterior to anterior compression force (arrow) can be applied on the fracture site by inserting the cortical screw through the proximal hole, with the equivalent vector applied by lag screw insertion (the ankle is internally rotated on the operative field to facilitate screw insertion).

**Figure 5:**
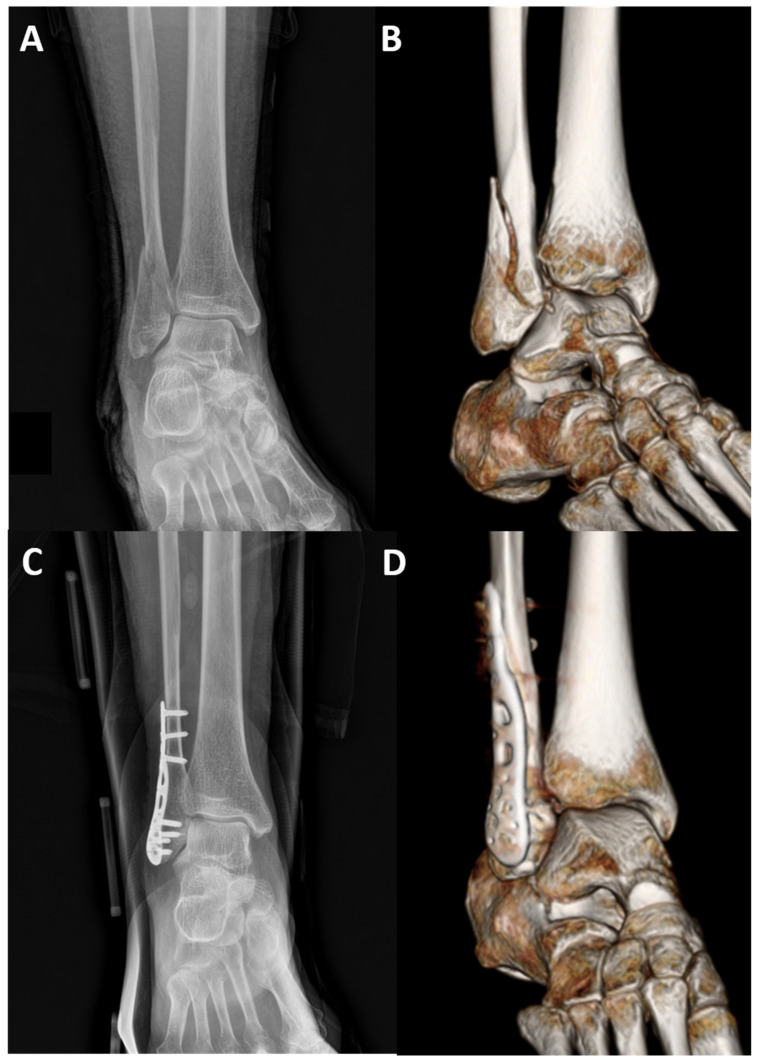
Preoperative radiograph (**A**) and CT (**B**) images of an isolated Weber B type fracture. Postoperative radiograph (**C**) and CT (**D**) images showing the fracture treated with a three-dimensionally pre-contoured locking plate that is properly fit on the bone.

As the proximal portion of the plate is bent posteriorly, posterior to anterior compression force can be applied on the fracture site by inserting the cortical screw through a proximal hole, with the equivalent vector applied by lag screw insertion (Figure 4). After reconfirming the intended location of the plate using a C-arm image intensifier, the Kirschner wires or reduction clamps were removed, and four remaining locking screws were fixed at the distal and proximal regions between the first two screws (Figure 5). 

### 2.2. Postoperative Management

Non-weight-bearing active range of motion (ROM) exercise was started immediately, and walking with crutches without weight bearing was allowed for 2 weeks with a short leg splint. After 2 weeks, the patients were allowed to walk with tolerable weight bearing without a splint or walking brace. At 4 weeks, the patients were gradually allowed to walk with full weight bearing in normal shoes and could resume normal activities as tolerated. At 8 weeks, sports activities were allowed.

### 2.3. Postoperative Evaluations

The functional and radiographic ratings of the patients at least 12 months after the surgery were evaluated according to the criteria reported by McLennan and Ungersma (Table 1) [12]. In addition, the interval to radiographic union was noted. The criteria for bony union were defined as the presence of a bridging bone in three out of four cortices in the radiographic images [13]. All the radiographic outcomes were evaluated by two orthopaedic surgeons who did not participate in the surgery. Then, the final decision of the radiographic finding was made based on the consensus between the two surgeons through detailed discussion. The incidence of postoperative complications, including metal failure, nerve irritation, peroneal tendinopathy, and infection, was evaluated through medical record reviews.

### 2.4. Statistical Analysis

For all variables, the Shapiro–Wilk normality test showed no evidence of a non-normal distribution. For comparison between two groups, a two-sample t-test and Fisher’s exact test were used. A *p* value < 0.05 was considered to be a significant difference. Data processing and statistical analyses were performed using R version 3.3.1 (Foundation for Statistical Computing, Vienna, Austria).

## 3. Results

The mean age (and standard deviation) of the included patients at the time of surgery was 49.68 ± 15.69 years (range 19 to 81 years). No significant differences in patient demographics were detected between the two groups (Table 2). The operation time was significantly shorter in Group B (Group A, 45.29 ± 8.98 min; Group B, 32.9 ± 9.73 min; *p* < 0.001). According to the McLennan and Ungersma criteria, all but two and three patients showed good or fair outcomes for the functional and radiographic outcomes, respectively. 

Comparing the two groups, Group B showed a better functional outcome (Group A, Good = 22, Fair = 19, Poor = 0; Group B, Good = 23, Fair = 5, and Poor = 3; *p* < 0.0046), while no difference between the two groups was found in terms of the radiographic outcomes (Group A, Good = 33, Fair = 8, Poor = 0; Group B, Good = 26, Fair = 3, and Poor = 2; *p* = 0.143). Likewise, the time to bony union was within 14 months in all patients, and there was no significant difference between the two groups (group A, 11.59 ± 1.5 months; group B, 10.71 ± 2.61 months; *p* = 0.0821). Lastly, there were no postoperative complications observed in either group (Table 3).

## 4. Discussion

In this study, isolated Weber B type fractures treated with the use of a three-dimensional anatomically pre-contoured lateral locking plate showed satisfactory functional and radiographic outcomes without any complications. In addition, cases treated only with the plate demonstrated shorter operation times, better functional outcomes, and more consistent radiographic outcomes compared with those treated with a lag screw and plate together. As a result, considering the morphology of distal fibula, our hypothesis that, with the use of the plate, the fracture site could be fixed properly regardless of interfragmentary lag screw fixation, was verified.

Anatomically, the fibular body presents four borders—the anterolateral, the anteromedial, the posterolateral, and the posteromedial. Among these, the anterolateral border of the fibula runs vertically downward starting from the top and then curves lateralward at the middle of the bone [14]. As a result, the lateral surface of the fibular body is not on the same plane with the lateral malleolus, and the appropriate lateral placement of the plate could become interrupted. 

In other words, the proximal portion of the two-dimensionally pre-contoured plate may not contact properly with the fibular body if it is adjusted to the lateral malleolus, which interrupts the proper placement of the proximal screws. In addition, a two-dimensionally pre-contoured plate may result in impingement between the plate and the bulky head of the lag screw inserted in advance [7].

Considering the anatomic characteristics of the distal fibula, we contrived a three-dimensionally pre-contoured plate with its proximal portion twisted posteriorly. In this way, the whole of the plate can be attached to both the lateral malleolus and lateral surface of the fibular body. In addition, the midportion of the plate, which generally overlaps with the lag screw inserted in advance, is placed far posterolaterally to the screw insertion site owing to its posteriorly twisted morphology. As a result, the plate can be used together with a lag screw without concerns about screw impingement.

Among different measures to place the plate in an isolated Weber B type fracture, posterior anti-glide plating is known to be advantageous over lateral plating because it prevents the distal oblique fibular fragment from gliding through the proximal fragment when axial forces are applied to the ankle [15]. Owing to the posteriorly twisted morphology, the plate used in this study may also effectively buttress the fragment. In addition, using a compression screw through the posteriorly twisted hole of the plate, posterior to anterior fixation provides compression force with the equivalent vector acquired through lag screw insertion. As a result, cases operated only with the plate demonstrated comparable postoperative outcomes compared with those with the plate and screw and were significantly decreased in operation time. Therefore, the three-dimensionally pre-contoured plate itself provided the advantages of both posterior anti-glide plates and lag screws.

Although only the isolated Weber B fractures without unstable syndesmotic injuries were included in this study, about half of Weber B malleolar fractures are reportedly combined with distal tibiofibular syndesmosis injuries [16,17,18]. After the fracture fixation, syndesmotic instability was routinely evaluated intraoperatively through the fluoroscopic lateral stress test in our institution. If the stress test turned out to be positive, either a cortical screw or Tightrope was inserted through the plate hole at 1 cm proximal to the syndesmotic level. As this hole is usually located on the posteriorly twisted midportion of the plate, it was advantageous to retain the ideal trajectory of the fixation, which was recently found to be around 18.8 degrees relative to the ground level [19]. Following research to validate whether the three-dimensionally pre-contoured plate is favourable to the two-dimensionally pre-contoured plate in retaining the ideal trajectory of syndesmotic fixation should be performed next.

In this study, no complications were found postoperatively. Previous studies have demonstrated injuries or irritations to surrounding structures in ankle fractures treated surgically, for instance, the superficial peroneal nerve (SPN) or the peroneal tendons. Since dorsal cutaneous branches of the SPN course close to the distal fibula and may cross it, it is vulnerable to injury, especially in lateral plating [20]. Indeed, damage to the SPN is known to be responsible for painful symptoms and lower functional outcomes. 

In addition, peroneal tendinopathy has been reported in plates placed in an anti-glide manner, posterior to the fibula, which significantly improved after plate removal [21]. Also, it is expected that the twisted nature of the three-dimensionally pre-contoured plate was effective in avoiding the surrounding structures with the bridging midportion of the plate keeping distance from the course of the SPN and only the proximal portion of the plate placed posteriorly to the distal fibula.

This study is limited owing to its non-randomized, retrospective nature with a small sample size of 72 cases and short-term follow-up. In addition, the study may have demonstrated a higher level of evidence if the three-dimensionally pre-contoured plate was compared with a two-dimensionally pre-contoured plate. A follow-up prospective comparative study that involves sufficient cases and randomization would strengthen the validity of this study. Only McLennana and Ungersma’s criteria were used to evaluate the functional outcomes. Additional scoring systems may have validated the outcomes of this study more precisely.

## 5. Conclusions

In conclusion, the use of a three-dimensionally pre-contoured lateral locking plate fixation for isolated Weber B type fractures demonstrated satisfactory functional and radiographic outcomes, regardless of lag screw insertion. We suggest that the plate itself can be used effectively without a lag screw, thus, contributing to stable fixation, a short operation time, and lower postoperative complications.

## Figures and Tables

**Table 1:** Functional and radiographic rating according to McLennan and Ungersma’s criteria (compared to the normal ankle).

Functional	
Good	80% of normal strength and range of motion (ROM) without pain or stiffness, return to previous activity level
Fair	>60% of normal and ROM without stiffness, occasional pain following activity
Poor	<60% of normal strength and ROM, with pain and stiffness at rest
Radiographic	
Good	Fibula out to length
	<2 mm posterior displacement in the groove of the tibia
	<1 mm increase in the medial clear space
Fair	Fibula shortened ≤ 2 mm
	2–4 mm posterior displacement
	1–3 mm increase in medial clear space
Poor	Fibula shortened > 2 mm
	>4 mm posterior displacement
	>2 mm lateral displacement
	>3 mm increase in medial clear space

**Table 2:** Patient demographics.

	Group A	Group B	*p* Value
	(*n* = 41)	(*n* = 31)	
Age, year	47.9 ± 16.15	52.03 ± 14.98	0.2668
Sex			
Female	21	14	0.6414
Male	20	17	
BMI (m/kg^2^)	23.77 ± 3.2	24.56 ± 3.47	0.3301
Follow-up (months)	13.94 ± 1.58	13.39 ± 1.98	0.2093

BMI = body mass index.

**Table 3:** Comparison of outcomes between two groups according to use of the lag screw.

	Group A	Group B	*p* Value
	(*n* = 41)	(*n* = 31)	
McLennan and Ungersma criteria			
Functional rating			0.0046
Good	22	23	
Fair	19	5	
Poor	0	3	
Radiographic rating			0.143
Good	33	26	
Fair	8	3	
Poor	0	2	
Time to bony union	11.59 ± 1.5	10.71 ± 2.61	0.0821
Operation time (min)	45.29 ± 8.98	32.9 ± 9.73	<0.001
Complication	0	0	

## Data Availability

The datasets used and/or analysed during the current study are available from the corresponding author on reasonable request.
